# Integral Valorization of *Posidonia oceanica* Balls: An Abundant and Potential Biomass

**DOI:** 10.3390/polym16010164

**Published:** 2024-01-04

**Authors:** Rim Mnafki, Amaia Morales, Leyre Sillero, Ramzi Khiari, Younes Moussaoui, Jalel Labidi

**Affiliations:** 1 Organic Chemistry Laboratory (LR17ES08), Faculty of Sciences of Sfax, Sfax 3018, Tunisia; 2 Faculty of Sciences of Gafsa, University of Gafsa, Gafsa 2112, Tunisia; 3 Biorefinery Processes Research Group, Department of Chemical and Environmental Engineering, University of Basque Country (UPV/EHU), 20018 Donostia-San Sebastian, Spain; 4 Biorefinery Processes Research Group, Department of Chemical and Environmental Engineering, University of Basque Country (UPV/EHU), 01006 Vitoria-Gasteiz, Spain; 5 Department of Textile, Higher Institute of Technological Studies (ISET) of Ksar-Hellal, Ksar-Hellal 5070, Tunisia; 6 CNRS, Grenoble INP, LGP2, University of Grenoble Alpes, 38000 Grenoble, France

**Keywords:** *Posidonia oceanica*, lignin, cellulose nanofibers, biorefinery

## Abstract

*Posidonia oceanica* balls (POB), a kind of seagrass, are a significant environmental issue since they are annually discharged onto beaches. Their current usefulness limits interest in their management and enhances the environmental problem. Therefore, in this research, the potential of this lignocellulosic biomass was studied from a holistic biorefinery point of view. To this end, an in-depth study was carried out to select the best pathway for the integral valorization of POBs. First, an autohydrolysis process was studied for the recovery of oligosaccharides. Then, a delignification stage was applied, where, in addition to studying different delignification methods, the influence of the autohydrolysis pre-treatment was also investigated. Finally, cellulose nanofibers (CNFs) were obtained through a chemo-mechanical treatment. The results showed that autohydrolysis not only improved the delignification process and its products, but also allowed the hemicelluloses to be valorized. Acetoformosolv delignification proved to be the most successful in terms of lignin and cellulose properties. However, alkaline delignification was able to extract the highest amount of lignin with low purity. CNFs were also successfully produced from bleached solids. Therefore, the potential of POB as a feedstock for a biorefinery was confirmed, and the pathway should be chosen according to the requirements of the desired end products.

## 1. Introduction

*Posidonia oceanica* (L.) is a slow-growing aquatic plant endemic to the Mediterranean basin, which is sometimes wrongly considered an algae [1]. This annual plant, which forms huge underwater meadows on the Mediterranean seabed, is capable of generating enormous quantities of leaf debris, roots and rhizomes that can be deposited as sediments, or can even reach the coasts due to currents and sea waves [2]. The leaves of *Posidonia oceanica* (PO) are made up of blades and sheaths. The blades fall off the plant annually, while the sheaths usually remain attached to the rhizomes. Therefore, on the one hand, leaf debris is generated and reaches the coasts, forming banks [3,4]; on the other hand, the rhizome fragments (with the sheaths), as well as the roots, can be entangled to form balls that will reach the coasts due to the movement of the waves [3,4]. These dried fibrous balls are known as PO egagropili.

*Posidonia oceanica* creates vast underwater meadows that encompass approximately 1.5% of the Mediterranean basin and involve 16 different Mediterranean nations, where it plays a crucial role in the dynamics of the coastal ecology [5]. The Tunisian coasts have significant accumulation of *P. oceanica* sea balls (POB), which requires summer beach cleanings. Since these wastes have to be regularly disposed of, and their cost is not negligible, there is a clear necessity to find a way to valorize these lignocellulosic residues that currently have no added-value applications. An appropriate solution to this issue could be the integral valorization of this readily available and renewable lignocellulosic biomass as a source of potential bio-based products [6].

With the urgent need for alternatives to fossil resources, research academics from all around the world are becoming increasingly interested in lignocellulosic biomass [6], owing to its abundance, availability and renewable nature [7,8]. It is mainly composed of three biopolymers (cellulose, hemicelluloses and lignin) combined in an elaborate and complicated architecture. POB can be a feasible alternative since they are widely available in large quantities in most of the Mediterranean countries [9]. Therefore, POB can be considered as lignocellulosic biomass with great potential for valorization through biorefinery processes, as has been already evidenced by various studies, in which POB have been used for the extraction of bioactive compounds, carboxymethylated cellulose production and as biosorbents [10,11,12].

In this new global context, biorefineries are expected to be the solution to many environmental problems. According to the International Energy Agency (IEA) definition: “Biorefinery is the sustainable processing of biomass into a spectrum of marketable bio-based products and bioenergy.” [13]. In other words, a biorefinery is based on the same principles as a petroleum refinery, with the objective to obtain various products such as chemicals, bioenergy and biofuels from a single feedstock using integrated production [14]. In the case of biorefineries, these feedstocks can be widely varied; nevertheless, it is important to choose the appropriate biomass to be used for an economically profitable process. Therefore, available, abundant and low-cost feedstocks are sought, with lignocellulosic biomass being one of the most promising. In terms of sustainable biorefinery, the correct selection of the processes to be integrated in the biorefinery is necessary, taking into account the social, environmental and economic perspective [15].

Pre-treatments are essential steps to adjust the chemical composition, increase the processability and open up the internal structure of raw lignocellulosic biomass that will be used in various conversion processes [16]. In terms of cost and environmental impact, the autohydrolysis approach for the pre-treatment of lignocellulosic materials is interesting, since it only uses water at subcritical conditions and does not require additional chemicals [17]. Another important pre-treatment for the fractionation of lignocellulosic biomass is the delignification. Some of the most promising techniques are based on organosolv pulping, where the lignocellulosic biomass is usually treated in the presence of a combination of water and organic solvent(s), and sometimes also with a low concentration of an acid catalyst (organic or inorganic). Alkaline delignification is another widely used the delignification process, such as organosolv, which uses aqueous mixtures of alkaline compounds, with KOH and NaOH being the most commonly used reactives. The delignification process may lead to three separate streams: a cellulose-rich pulp, a lignin-rich solid precipitate and a hemicellulose-rich liquid, the composition of which will depend on the previous pre-treatment [18]. 

Recently, there is an increasing interest in the use of low boiling carboxylic acid mixtures, such as acetic acid or formic acid, as organic solvents for the organosolv delignification process, since this technology can operate at relatively low temperatures and pressures [19]. On the one hand, the use of water/carboxylic acid mixtures has been studied. An example of this is the study conducted by Pham et al. (2022), which reported the influence of various parameters on the delignification process of sugarcane bagasse using an aqueous formic acid mixture [20]. In this work, a delignification yield of more than 90% was achieved with a glucan content of more than 92%. For this purpose, a 90 min process at 130 °C with an 80% formic acid mixture was used. In the work developed by Linan et al. (2023), the use of an aqueous mixture of acetic acid and H_2_SO_4_ as a catalyst for the delignification of different acai berry bagasse residues was investigated [21]. In this work, it was concluded that the necessary conditions to obtain the highest-purity lignin did not correspond to the conditions to obtain the highest delignification yield. The best extraction yields were obtained at low times and high acid concentrations (92.5% acid and 1 h), while longer times (up to 5 h) were necessary to obtain the highest purity. On the other hand, the combined use of various carboxylic acids in aqueous mixtures has also been studied, although to a lesser extent. An example of this is the work performed by Labauze et al. (2022), where they described the optimization of the process carried out to obtain lignin from wheat straw using a mixture of formic acid/acetic acid/water [22]. The highest yield was reached by operating at 105 °C for 2 h and 30 min, recovering up to 73% of the lignin. The use of these low-boiling organic compounds also allows them to be easily recovered and subsequently reused.

Lignin, due to its properties, has a wide range of applications, ranging from precursor of several high added-value chemical products, to its use in the production of bio-materials or even biofuels [23,24]. Hemicelluloses, among other possible applications, can be used to obtain different oligosaccharides with diverse applications [25]. Meanwhile, cellulose could be converted into bioethanol [26]. It could also be used to produce various bio-materials [27], where cellulose nanofibers (CNF) are one of the most promising options.

As aforementioned, POB has been used for its fractionation via different biorefinery processes. For instance, some authors produced nanocrystalline cellulose from POB via sodium chlorite and acetic acid delignification, usually known as the bleaching process. In this way, the authors also recovered the liquid phase, rich in lignin, and purified the cellulose before acid hydrolysis [3]. Other authors also extracted cellulose from POB using alkaline delignification and a bleaching step with sodium hypochlorite [11]. Nevertheless, most of the published works aim to valorize mainly one of the fractions of this biomass.

From a circular economy standpoint, the goal of this work was to develop an integrated biorefinery using POB waste. To do so, the recovery of oligosaccharides from different autohydrolysis pre-treatments was firstly investigated. Then, lignin was extracted employing several delignification methods, studying the influence of the pre-treatment on the obtained products. Finally, cellulose nanofibers (CNFs) were produced from the delignified solids with and without a bleaching step. In addition, in this work, a discussion of the effects of each of the stages on the resulting products is provided in order to identify the best biorefinery to enhance the green and circular economy.

## 2. Materials and Methods

### 2.1. Raw Material and Chemicals

The *Posidonia oceanica* balls (POB) were collected from Chatt-Mariem Sousse (Tunisia) in August 2022. This waste was washed with water several times to eliminate sand and dirt. Then, the raw material was dried under room temperature, ground in a laboratory grinder and sieved to obtain fibers with a size of <4 mm.

Toluene and dimethylformamide were supplied by Thermo Fisher Scientific Inc. (Waltham, MA, USA). Panreac Química S.L.U. (Barcelona, Spain) provided sulphuric acid, acetic acid (glacial) technical grade, sodium chlorite and sodium hydroxide. Ethanol absolute (synthesis grade) was supplied by Scharlab S.L. (Barcelona, Spain).

### 2.2. Autohydrolysis Process

The autohydrolysis pre-treatment was performed in a high-pressure stirred reactor (1.5 L stainless steel 5100 Parr reactor with a 4848 Parr controller (Parr Instrument Company, Moline, IL, USA). The non-isothermal autohydrolysis treatment was conducted with water at 130 °C, 150 °C and 180 °C, employing a solid/liquid ratio (SLR) of 1:8 (*w*/*w*). These conditions were selected based on a previous work [28]. When the reaction temperature was reached, the reactor was cooled down and the liquid and solid phases were separated by vacuum filtration. The liquid phase was recovered for analysis, and the solid phase was washed with distilled water until neutral pH and dried at room temperature. This resulted in the 3 autohydrolyzed solids to be compared, which were named as: autohydrolyzed solid at 130 °C (A130), autohydrolyzed solid at 150 °C (A150), and autohydrolyzed solid at 180 °C (A180).

### 2.3. Delignification Processes

The delignification was carried out using the solid obtained after autohydrolysis at 180 °C (A180), and the non-autohydrolyzed raw material. Two different processes, acetoformosolv and alkaline processes, were studied in this step. Both processes were conducted in an autoclave (VWR International S.r.l., Milano, Italy) at 121 °C for 90 min. For the acetoformosolv process Acetic-Acid/Formic-Acid/Water (30/60/10 (*v*/*v*/*v*)) mixture, and SLR of 1:10 (*w*/*v*) was employed based on the conditions reported elsewhere [29]. The alkali process used 5 wt% sodium hydroxide solution, with an SLR of 1:10 (*w*/*v*).

After the delignification, the mixture was separated by vacuum filtration. The solid phase, after its cleaning with water, was oven-dried for 24 h at 50 °C to obtain three delignified solids, named: alkali delignified raw material (D-NaOH), acetoformosolv delignified raw material (D-AFW) and acetoformosolv delignified autohydrolyzed (180 °C) raw material (D-A180-AFW).

Lignin precipitation from the liquid fraction was conducted by two different methods depending on the delignification process employed. For the liquid fraction derived from the acetoformosolv delignification, the lignin was precipitated by the addition of 5 volumes of distilled water. Two different lignins were obtained from this process, the lignin derived from the previously autohydrolyzed raw material (L-A180-AFW) and the lignin derived from the raw material without pre-treatment (L-AFW). For the precipitation of the lignin from the liquor obtained in the alkaline delignification, H_2_SO_4_ was added until a pH of 2 was reached. The lignin obtained by this process was named as L-NaOH.

### 2.4. Cellulose Nanofiber (CNF) Production

The delignified solids were firstly subjected to a bleaching treatment and, subsequently, to a mechanical treatment to obtain nanofibers. The bleaching was conducted using a reflux set-up, in an oil bath at 75 °C for 4 h, employing an aqueous mixture of acetic acid and sodium chlorite as reported by Morales et al. (2020) [30]. Then, the solid was recovered by filtration and washed with hot water until a neutral pH. After 24 h of drying at 50 °C, three bleached solids were obtained: bleached acetoformosolv delignified raw material (B-D-AFW), bleached acetoformosolv delignified autohydrolyzed raw material (B-D-A180-AFW), and bleached alkali delignified raw material (B-D-NaOH). 

Then, the cellulose obtained after bleaching underwent a mechanical treatment using a homogenizer to obtain the nanofibers. For this purpose, the bleached solids were dispersed in water using an Ultra-Turrax (Heidolph Instruments GmbH & Co. KG., Schwabach, Germany) for 15 min at 2000 rpm. Once this pre-treatment was finished, the mixture was subjected to a mechanical homogenization process, using a homogenizer (GEA Germany Düsseldorf, Düsseldorf, Germany) with 30 cycles, increasing the pressure progressively from 0 bar until 900 bar. The resulting nanofibers were classified as follows: CNF from bleached delignified autohydrolyzed raw material (CNF-A180-AFW), CNF from bleached acetoformosolv delignified raw material (CNF-AFW), and CNF from bleached alkali delignified raw material (CNF-NaOH).

### 2.5. Characterization Methods

#### 2.5.1. Solids and Liquid Phases Chemical Characterization

The chemical characterization of the raw material was carried out by measuring the extractives, ash, glucan, hemicelluloses and lignin content. The extractive compounds were evaluated in ethanol-toluene (TAPPI T204 cm-97). Ash content was measured using the TAPPI T211 om-02 standard procedure and moisture content employing TAPPI T264-om-88. Lignin, hemicelluloses and cellulose content, measured as glucan, were determined using NREL-TP-510-42618 [30]. 

The solids derived from the different biorefinery steps were chemically characterized by quantitative acid hydrolysis, following the procedure described in NREL-TP-510-42618. The chemical characterization of the liquid phases from autohydrolysis was carried out using HPLC (Jasco LC-Net II/ADC, 300 mm × 7.8 mm Aminex HPX-87H column) (Jasco-Spain, Madrid, Spain). Autohydrolysis was followed by a post-hydrolysis for each liquid obtained to ensure that only those polysaccharides were removed from the starting material during autohydrolysis. Post-hydrolysis and subsequent HPLC analysis was carried out following the methodology described in a previous work [30].

#### 2.5.2. Products General Characterization

Attenuated Total Reflectance-Fourier Transform Infrared Spectroscopy (ATR-FTIR) was employed to understand the chemical structure not only of the different solids (POB, A130, A150, A180, D-NaOH, D-A180, D-AFW, B-D-NaOH, B-D-AFW and B-D-A180-AFW), but also of the lignins (L-A180, L-AFW and L-NaOH) and CNFs (CNF-NaOH, CNF-AFW, CNF-A180-AFW) [30]. 

The purity of the obtained lignins (L-A180-AFW, L-AFW and L-NaOH) was also analyzed by quantitative acid hydrolysis, and Gel Permeation Chromatography (GPC) technique was employed to determine their weigh and number-average molecular weights, as well as their polydispersity indexes as described by Morales et al. (2018) [31].

The CNFs (CNF-NaOH, CNF-AFW, CNF-A180-AFW) were characterized by X-Ray Diffraction (XRD) and Atomic Force Microscopy (AFM) to determine their crystallinity and surface morphology, respectively, following the methodologies described in a previous work [30].

## 3. Results and Discussion

### 3.1. Raw Material Characterization

The chemical characterization of POB has been completed, from which it was concluded that this raw material was composed of 8.6 wt% of ash, 4.3 wt% of extractives, 34.2 wt% of lignin (32.6 wt% of insoluble lignin and 1.5 wt% of soluble lignin), 21.1 wt% of glucan and 18.1 wt% of hemicelluloses (measured as the sum of arabinosyl and xylan substituents, and acetyl groups). Even though the balls were carefully cleaned before analysis, the maritime environment and the sand contamination of the balls are to blame for the significant amount of ash (approximately 9%). This value, however, was lower than that reported by other authors for the same raw material, who reported 12% of ash [9,32]. Same applies to the extractable content, although in this case the difference was bigger, reporting values of between 7 and 11%. Regarding the acid-insoluble lignin content, the lignin content calculated in this work was higher than that reported by other authors, in particular the 24% reported by Pilavtepe et al. (2013) [33]. However, in the case of hemicelluloses, the values were variable in previous studies, with the measured value here within this range (13–22%) [9,33].

### 3.2. Autohydrolysis

Once the chemical composition of the raw material was known, and verifying that it had a high hemicelluloses content, the use of a pre-treatment prior to delignification to obtain oligosaccharides in addition to lignin and cellulose was considered to be of great interest. Therefore, the use of autohydrolysis as a pre-treatment before the delignification stage was proposed. To our knowledge, there were no previous studies about POB using autohydrolysis; therefore, it was decided to conduct a preliminary study for the selection of the best reaction conditions in order to extract oligosaccharides. 

Three different operating temperatures were considered in this work in order to select the best one. As shown in Table 1, it was noticed that, as expected, the increase in temperature resulted in a higher solubility of the solid. In particular, when the reaction temperature grew from 130 to 180 °C, the solubility yield increased from 8.76 to 18.46%. The latest conditions also permitted the achievement of the best results in the extraction of hemicelluloses from the raw material, with the arabinan groups being the most extracted (Table 1). According to these results (Table 1), the autohydrolysis treatment carried out at 180 °C was the most efficient pre-treatment of all, as it had the highest solubility (18%) and the highest amount of monosaccharide eliminations (xylose, glucose and arabinose). It was therefore concluded that the best conditions for autohydrolysis were: 180 °C, SLR of 1:8 (*w*/*w*) under non-isothermal conditions. However, the results obtained in the pre-treatment of this feedstock were lower than those reported for other lignocellulosic biomasses. For instance, Morales et al. (2020) reported a solubility of 40% for almond shells using a process at 179 °C for 23 min [30]. A few years earlier, Gullón et al. (2018) reported a solubilization range between 23 and 42% for the non-isothermal autohydrolysis process of chestnut shell [34].

The results showed that the autohydrolysis process was not very selective, as in addition solubilizing hemicelluloses, there was also solubilization of a small part of the lignin, and probably of other compounds from the extractable fraction, which is usual [35]. A more in-depth analysis of the composition of the autohydrolysis liquor under best conditions showed that there was a higher solubility of monosaccharides (6.1 g/L) than oligosaccharides (0.7 g/L). This could be attributed to the degradation of oligosaccharides due to the severity of the process, so it would be necessary to study this parameter in the future. However, autohydrolysis is not only used to obtain mono- or oligosaccharides, it is also a pre-treatment that allows the structure of the lignocellulosic biomass to be prepared to make it more accessible for further processing in the biorefinery waterfall [36]. Thus, this is expected to allow lignin and cellulose to be obtained more easily, with higher purity and better properties, just as it has been achieved in the case of other previously studied biomasses, such as wheat straw [37,38] or almond shells [30].

### 3.3. Delignification

The delignification treatment led to higher solubility than the autohydrolysis process. The highest solubility was obtained in the direct delignification process using AFW from the starting raw material, followed by NaOH delignification. Delignification after autohydrolysis had lower solubility, but it should be noted that solubilization of part of the main POB components had already happened in the previous stage. 

According to the results shown in Table 1, delignification with NaOH (D-NaOH) eliminated the highest amount of lignin with almost 64%. This process also had the highest glucan removal rate. Delignification with AFW after autohydrolysis (D-A180-AFW) generated the lowest lignin removal, with the highest hemicelluloses elimination. These results compared with those obtained by direct AFW delignification (D-AFW) revealed that this process resulted in a higher lignin and glucan removal, while the difference in the hemicelluloses was less significant. 

The solid composition and lignin yields obtained for each example were anticipated to vary since the methods of delignification involved in each procedure (acetoformosolv or alkaline) were different. Analyzing the chemical composition of the delignified solids in more detail (Table 2), it was seen that there were no significant variations in lignin content in D-A180-AFW and D-AFW. However, studying the glucan content, it is clear that autohydrolysis prior to delignification allowed obtaining solids richer in cellulose and with a lower amount of impurities, such as hemicelluloses. This could be due to the effect of the pre-treatment on the lignocellulosic structure of the biomass, which, once opened, facilitated the extraction of the hemicelluloses. The D-NaOH had the lowest amount of glucan and the highest amount of impurities. Therefore, it can be said that the use of autohydrolysis followed by delignification with organic acids promoted the production of a solid with a higher percentage of glucan. This is in line with the results reported by del Rio et al. (2020) in the study of Paulownia wood biomass biorefinery [39]. In this work, the authors also concluded that the combination of autohydrolysis with delignification, in this case by organosolv process, provided the best alternative.

In the work carried out by Morales et al. (2021) for the valorization of walnut shells, the proposed integral biorefinery process managed to obtain a solid richer in cellulose, but with a similar hemicelluloses content, and a lower lignin fraction, in comparison with sample D-A180-AFW [40]. Solids with glucan contents above 70% were achieved in the delignification carried out by Dominguez et al. (2020) using formosolv for the treatment of Paulownia wood, which is considerably higher than the results reported in this work [41]. This difference may be due, on the one hand, to differences in the composition and structure of the treated feedstock, and on the other hand, to the employed processing conditions. Regarding the alkaline treatment, in the optimization study performed by Morales et al. (2018) for the chestnut shells’ delignification process, the solubility achieved (31%) was similar to the one reported in this work [31]. However, concerning the composition of the solid, the glucan content reported was 60%, while in this work it did not even reach 30%. This suggests the importance of optimizing the process for each raw material.

### 3.4. Bleaching

The bleaching stage conducted before CNF was found to be a necessary step for the subsequent production of CNF using mechanical treatment, according to the experimental results that are not shown in this work. This is in line with the statement by Agwuncha et al. (2019), who explains that it is necessary to remove as much lignin and hemicellulose as possible, which act as glue in the lignocellulosic structure, in order to obtain a high-quality cellulose for the subsequent production of CNF [42]. In this work, bleaching may be necessary due to the fact that the conditions used in the autohydrolysis and delignification treatments were not aggressive enough, which means that the lignocellulosic structure could not be suitable to obtain CNF under the tested conditions.

In accordance with the results presented in Table 2, the bleaching process had different effects depending on each treated solid. Thus, it was observed that the bleaching performed to the delignified solids D-A180-AFW and D-NaOH managed to eliminate almost all the remaining lignin in the solid, with lignin percentages of 0.13% and 1.54% for B-D-A180-AFW and D-NaOH, respectively. These results are in line with those published by Morales et al. (2020), where the same bleaching process was conducted on alkaline and organosolv delignified almond shells [30]. The solid B-D-A180-AFW had a lignin content of more than 22%, which leads to the conclusion that processing conditions were not suitable. This also confirms the importance of the autohydrolysis pre-treatment in the production of high-purity cellulose.

The B-D-A180-AFW was the richest in cellulose of the three solids, as well as the one with the lowest impurities, both lignin and hemicelluloses. Comparing the solids resulting from the other two processes (B-D-AFW and D-NaOH), it can be observed that the differences in glucan and hemicellulose content were not very significant. Therefore, it is confirmed that the complete biorefinery made up of the processes of autohydrolysis, delignification with organic acids and bleaching is the best option for the valorization of the three main fractions of POB, although these results must be confirmed by product characterization.

### 3.5. Characterization of the Resulting Fractions

#### 3.5.1. Resulting Solids from Each Treatment 

**Figure 1:**
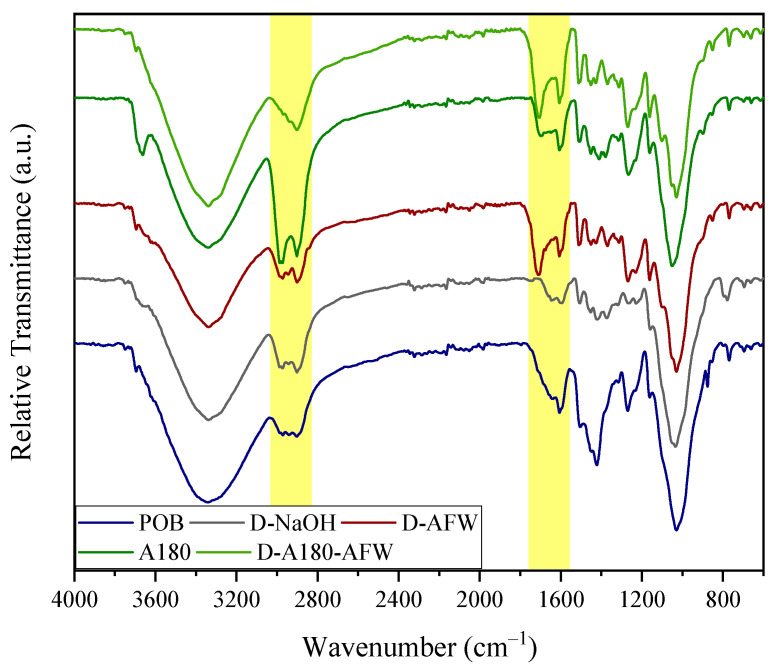
FTIR spectra of the raw material, autohydrolyzed solid and delignified solids.

In order to examine the modifications to the solids after the performed treatments, the FTIR spectra of POB, A180, D-NaOH, D-AFW and D-A180-AFW were collected (see Figure 1).

The FTIR spectra of all the samples showed the typical bands of lignocellulosic material, with some differences between them. In the range 2800–3600 cm^−1^, the band related to –OH stretching vibrations remained similar (3400 cm^−1^), the signal associated with stretching vibrations of –CH groups of cellulose and lignin (2890–2990 cm^−1^) were intensified, especially in the case of the A180 and D-A180-AFW samples, probably due to hemicellulosic removal [30]. The next significant variations were observed in the range 1600–1710 cm^−1^, which were related to the acetylation reaction during this acetoformosolv delignification method [43]. The diminution on the intensity of the signals at approximately 1510 and 1230 cm^−1^ in all samples, which are characteristic of C–C aromatic skeletal vibration and =C–O–C axial asymmetric strain, suggested that both hemicelluloses and lignin were removed [30,44]. The peak at approximately 1420 cm^−1^ in the spectrum of the raw material presented a significant reduction after any treatment. This signal can be either related to CH_2_ scissor motion in cellulose [45] or to aromatic skeleton vibrations in lignin [46], indicating their degradation as the peak was reduced. Nevertheless, the slight intensification of the weak signals at approximately 1320, 1160, 1110 and 1050 cm^−1^ associated with O–H bending, C–O–C glycoside bonds asymmetrical stretching, C–OH stretching and C–O–C pyranose ring vibration, subsequently, suggested that the presence of cellulose was more evident after the employed chemical treatments [44].

#### 3.5.2. Biorefinery Products 

##### Lignin Characterization

As aforementioned, quantitative acid hydrolysis was used to measure the purity of the lignin samples (see Table 3), which permitted to check the selectivity of the lignin extraction techniques. 

As shown in Table 3, the purity values were higher for acetoformosolv lignins than for alkaline lignins. In fact, the acetoformosolv lignins presented higher purities than 80% (83.5 and 92.0% for L-AFW and L-A180-AFW, respectively), whereas the alkaline lignin had a purity close to a 70%. These results confirmed that the selectivity of acetoformosolv extractions was higher than the one for alkaline process. A similar trend was reported by Morales et al. (2022), in which organosolv lignins from almond and walnut shells also presented higher purities than alkaline ones [47]. However, the purities for alkaline lignins reported by these authors (50–60%) were much lower than the ones for L-NaOH in this work. Additionally, it was found that the purity of the acetoformosolv lignin with a previous autohydrolysis treatment (L-A180-AFW) was significantly higher than the one for the acetoformosolv lignin without pre-treatment (L-AFW). This fact suggested that autohydrolysis permitted an effective removal of hemicelluloses in POB, significantly increasing the purity of the recovered lignin. Similar results were reported by other authors for lignins extracted from almond and walnut shells with and without a hydrothermal pre-treatment [47]. Nevertheless, the increase reported in their case (≈5%) was lower than the one for POB acetoformosolv lignin in the present work. This fact evidenced the importance of performing a pre-treatment.

As shown in Table 3, GPC analyses of L-NaOH, L-AFW and L-A180-AFW isolated from *P. oceanica* led to the estimation of their number-average (M_n_), weight-average (M_w_) molecular weights and polydispersity indexes (M_w_/M_n_). According to the values displayed in Table 3, the lignins presented weight-average molecular weights between 10,100 and 11,550 g/mol and number-average molecular weights in the range of 1600–3300 g/mol. Among them, L-NaOH was the one differing the most, presenting the lowest number-average molecular weight value and a M_w_ of approximately 11,000 g/mol. These values led to a high polydispersity index (≈6.7), indicating the heterogeneity of this sample. These results are aligned with the trends reported by other authors [30,47], from which it can be clearly concluded that alkaline processes result in more heterogeneous lignins, probably due to the degradation of high molecular weight lignin fractions and the repolymerization of some of them during the treatment [30]. These GPC results were slightly different to the ones reported by Rencoret et al. for POB lignins [48], which presented lower weight-average and number-average molecular weights (6100 g/mol and 2740 g/mol, respectively), leading to a more homogeneous lignin (M_w_/M_n_ = 2.2). Nevertheless, it should be noted that the employed isolation was performed via ball milling and an extraction with dioxane-water, which could have affected the molecular weight.

**Figure 2:**
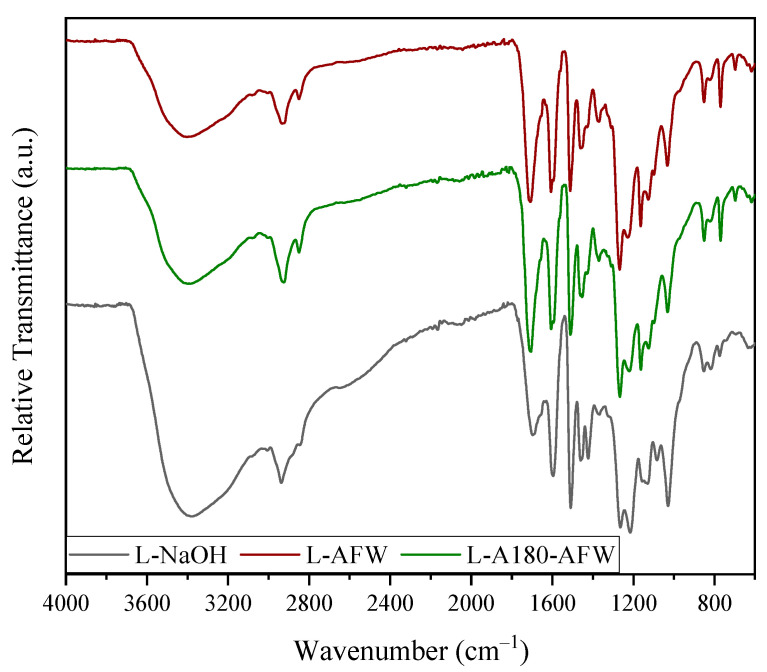
ATR-FTIR spectra of the obtained three lignins.

The determination of the chemical structure of the lignins obtained was performed by FTIR, as shown in Figure 2. As can be appreciated, the three samples presented the characteristic peaks of lignin in their spectra, with the L-NaOH sample being the most different. 

The three samples presented the typical absorption band at 3400 cm^−1^ corresponding to the –OH stretching vibrations, as well as the signals associated with stretching vibrations of the C–H (CH_3_, CH_2_ and CH) of lignin and polysaccharides. The peak at approximately 2850 cm^−1^ was less prominent for the L-NaOH sample, probably due to the presence of hemicellulosic as reported by Morales et al. (2022) for alkaline lignins [47]. The next notable change was observed at approximately 1700 cm^−1^, which was attributed to C=O stretching vibration in unconjugated ketone, carbonyl and ester groups [46]. This peak was more intense in the acetoformosolv lignin spectra, probably due to a higher acetyl group content derived from the acetylation reaction during this isolation method [43]. In the three samples, the characteristic peaks of aromatic skeletal vibrations were identified at approximately 1600, 1515 and 1425 cm^−1^, although the latter was more intense in the L-NaOH sample. The signal at 1373 cm^−1^ in the samples related to aliphatic C–H stretching in CH_3_ groups was less strong in the case of L-NaOH, which was also related to the acetylation of the lignins during acetoformosolv process [29,43]. The prominent band at 1270 cm^−1^ appearing in all samples was attributed to guaiacyl ring breathing with C–O stretching [29,46], and the signal at approximately 1130 cm^−1^ related to syringyl units was also identified [43,46]. Moreover, the peaks at 1080 and 1030 and 850 cm^−1^ corresponding to C–O deformation in secondary alcohols and aliphatic ethers, and aromatic C-H in-plane deformation plus C–O deformation in primary alcohols were detected [46], being more intense for L-NaOH probably due to esterification reactions in the case of organic acids. From these results, it was confirmed that the extraction method altered the structure of the obtained lignin, even if the modifications were not very significant. However, autohydrolysis did not seem to have a great effect on the isolated lignin, since both the L-AFW and L-A180-AFW samples presented almost identical ATR-FTIR spectra.

##### CNF Characterization

The last products to be characterized were the cellulose nanofibers (CNFs), which will be discussed in this section. For this characterization, FTIR, XRD and AFM were used. With the combination of these techniques, the effectiveness of the treatments to obtain CNFs from POB can be demonstrated.

The integrated biorefinery process proposed in this work included a bleaching stage of the cellulose-rich solids (B-D-NaOH, B-D-AFW and B-D-A180-AFW) prior to obtaining CNF, since it was concluded that, without bleaching, the process of obtaining CNF under the investigated conditions did not achieve the expected results. Nonetheless, some authors have previously considered cross-linked lignin to be a setback to fibrillating cellulose [49], which would explain these results.

**Figure 3:**
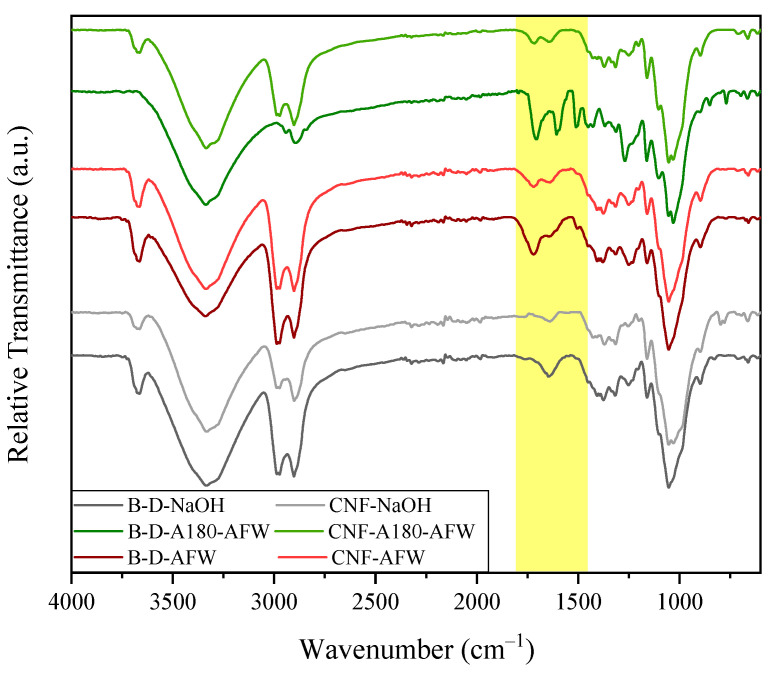
FTIR spectra of the bleached solids and the obtained CNF from bleached solids.

Figure 3 shows the results obtained from the FTIR characterization of the bleached solids (B-D-NaOH, B-D-AFW and B-D-A180-AFW), as well as the CNFs (CNF-A180-AFW, CNF-AFW and CNF-NaOH) obtained in this work. Compared to the delignified solids in Figure 1, both the bleached solids and the CNF presented narrower O–H bands at approximately 3350 cm^−1^, which could be related to the bleaching treatment [50] and the destruction of hydrogen bonds between the CNF due to the homogenization [51]. In addition, the bands in the range 2890–2990 cm^−1^, which are typical in cellulose structure [44], showed a significant intensification in the case of bleached solids and a lower intensification in the case of CNF. The diminution of the peak at 1510 cm^−1^ related to lignin removal was also appreciated in the three samples [44]. Apart from that, the small peaks at approximately 1320, 1160, 1105 and 1050 cm^−1^ related to O–H bending, C–O–C glycoside bonds asymmetrical stretching, C–OH stretching and C–O–C pyranose ring vibration were representative of the higher cellulosic exposure in the structure after the bleaching process [44]. What is more, a new peak also appeared at approximately 890 cm^−1^, which was attributed to the β–D–glucose group’s vibration [52].

Cellulose, due to its hydrogen bonds and Van der Waals forces, presents a crystalline structure, while hemicelluloses and lignin present an amorphous structure [53]. Therefore, the XRD technique was used to study how the different treatments affect the characteristics of the resulting CNFs. 

**Figure 4:**
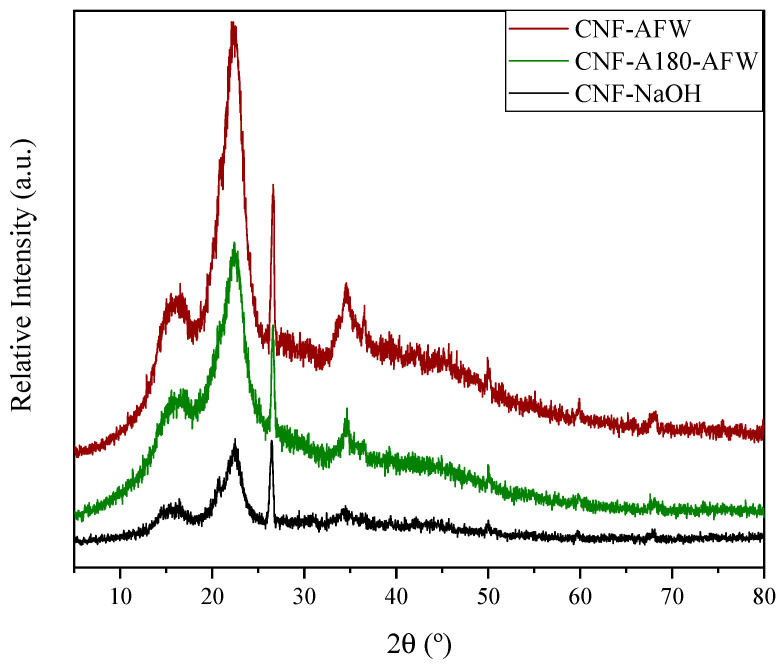
XRD diffractograms of different CNFs.

As seen in Figure 4, the diffractograms of the three CNF samples showed the characteristic peaks of cellulose [44,53]. These peaks were those appearing at approximately 16.2°, 22.4° and 34.5°, which were attributed to the (110), (002) and (004) crystallographic planes, respectively [44,54].

The CNF-AFW sample exhibited the highest intensity in the peaks belonging to the aforementioned crystallographic planes, suggesting that its crystallinity was superior. Sample CNF-A180-AFW showed the same pattern, although with a somewhat lower intensity. The lowest intensities were obtained with the alkaline treatment (CNF-NaOH). This might be due to the aggressiveness of the chosen treatments, which directly affect the crystallinity of the cellulose structure. Although high crystallinity peaks were expected for the samples with a high cellulose content, it is also possible that the non-crystalline regions of cellulose were disrupted due to the increase in the homogenization pressure, which also probably led to the destruction of part of the crystalline regions of CNFs and, consequently, to a decrease in crystallinity. These results were in accordance with those reported by Yao et al. (2023), who reported that the effect of the pressure during homogenization was much more significant than the number of applied cycles on the crystallinity of CNF samples [52]. What is more, lignin could have also acted as a shield during this process in the case of CNF-AFW [50].

In all the diffractograms, in addition to the already mentioned typical peaks, a peak of considerable intensity is observed at approximately 25°. This peak is associated with the presence of inorganic compounds, typical of sea-derived biomass, and is associated with the ash content of the starting biomass [54].

**Figure 5:**
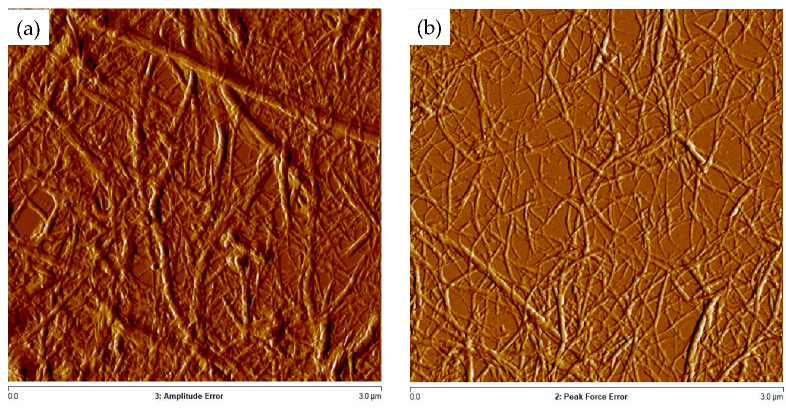
AFM images of (**a**) CNF-AFW and (**b**) CNF-A180-AFW.

The verification of CNF obtaining was performed by Atomic Force Microscopy analyses. As shown in Figure 5, CNF were clearly discerned for the CNF-AFW and CNF-A180-AFW samples, but not for CNF-NaOH. According to the width of the produced CNF, the ones from AFW process presented an average diameter of 66.38 ± 13.58 nm, whereas the ones from autohydrolyzed and delignified solid (CNF-A180-AFW) presented an average diameter of 47.26 ± 14.46 nm. These values were in the range of the ones obtained by Xu et al. (2018) for corn stover CNF after 30 passes at 500 bar (5–50 nm) [55]. The difference in the diameter size between both samples could be explained by the lignin content of their original bleached solids indicated in Table 2, since CNF-AFW were produced from a solid with still almost 23% of lignin, whereas CNF-A180-AFW were produced from a solid with only 1.5% of lignin. These results were in accordance with those reported by Trifol et al. (2021), who stated that lignin hampers the fibrillation efficiency and, therefore, wider fibers can be obtained [50]. 

### 3.6. Selection of the Most Suitable Process

The autohydrolysis of POB ensured the removal of a significant percentage of monosaccharides, in addition to facilitating the subsequent delignification process. This is in line with what was reported by Morales et al. (2020) in the integral biorefinery work carried out with almond shells [30]. Moreover, the obtained results confirmed the role of autohydrolysis for the production of high-purity lignins and CNFs with high crystallinity. Thus, the use of an integral biorefinery together with autohydrolysis would allow the valorization of the three most important fractions of lignocellulosic biomass, and more specifically of POB.

However, in the characterization of CNFs, it has been demonstrated that the products with the best characteristics were obtained by the single-step acetoformosolv delignification process. Therefore, if the main product to be obtained consisted of CNF, it could be interesting to save steps, which would also lead to a reduction in the economic cost of the process. These CNF could be employed as reinforcing agents in biocomposites, as reported by other authors [50]. This biorefinery would also allow the production of high-purity lignin, although the lignin yield would be lower than in the case of the other proposed biorefineries, according to the results regarding the composition of the delignified solids (Table 2).

Finally, it should be noted that the biorefinery process based on alkaline delignification led to a very interesting lignin yield. Nevertheless, even though the amount of lignin obtained was high, which could suggest that the process is cost-effective, the characterization of the lignins demonstrated that the product obtained had a low purity. Furthermore, the CNFs resulting from this biorefinery also showed characteristics that are far from current standards for these products. 

In conclusion, it has been confirmed that POB has the potential to be valorized using any of the proposed biorefineries in this work. Nonetheless, the selection of the best processes should ultimately rely on the products to be manufactured and the characteristics of these products.

## 4. Conclusions

The present study confirms, on the one hand, the potential of POB as a feedstock for its valorization by an integral biorefinery. On the other hand, it demonstrates the influence of different treatments on the final products (oligosaccharides, lignin and CNFs). The findings of this study reveal that the use of an autohydrolysis pre-treatment not only allows the hemicellulosic fraction to be recovered, but also favors the subsequent biorefinery processes resulting in the production of lignin and cellulose with better properties. The alkaline delignification process, although it achieved the highest lignin yield, led to final products with poorer quality, whereas the use of acetoformosolv delignification provided higher-purity lignin and better-quality CNFs. Furthermore, the necessity of the bleaching process for the production of CNFs under the presented working conditions was confirmed. Hence, it could be said that from the point of view of an integral biorefinery, the best biorefinery approach proposed in this study would be the one with the autohydrolysis pre-treatment followed by the acetoformosolv delignification step. Nevertheless, this decision should be taken according to the desired final products to be manufactured. In addition, the study of the economic impact of using a biorefinery with a greater number of stages and its possible economic and environmental benefits, as well as the optimization for the POB of each of the stages proposed in this biorefinery should be further studied in the future.

## Figures and Tables

**Table 1:** Solubility and removal rate of autohydrolysis and delignification treatments.

Treatment	Solubility (%)	Elimination (%)				
		Glucan	Xylan	Arabinan	Acetyl Groups	Klason Lignin
A130	8.76	0	0	0	0	28.79
A150	12.10	4.73	0	0	0	23.41
A180	18.46	10.82	15.40	47.36	0	28.93
D-A180-AFW	23.63	27.00	70.94	87.84	63.61	35.58
D-AFW	31.63	31.48	69.42	89.24	0	42.89
D-NaOH	29.44	34.22	29.59	26.86	70.74	63.81

**Table 2:** Chemical composition of the solids after the treatments: autohydrolysis, delignification and bleaching.

Treatment	Lignin (wt.%)	Glucan (wt.%)	Hemicellulose (wt.%)
A130	27.92 ± 1.13	27.91 ± 0.29	27.04 ± 0.47
A150	32.35 ± 0.71	26.04 ± 0.81	25.90 ± 0.28
A180	34.88 ± 0.75	28.33 ± 0.27	20.61 ± 0.38
D-A180-AFW	38.53 ± 0.91	35.46 ± 0.73	8.65 ± 0.06
D-AFW	39.87 ± 0.17	30.96 ± 0.22	9.52 ± 0.06
D-NaOH	23.72 ± 0.48	27.90 ± 0.58	25.63 ± 0.46
B-D-A180-AFW	0.13 ± 0.00	59.24 ± 1.47	8.03 ± 0.04
B-D-AFW	22.72 ± 0.62	49.33 ± 1.80	17.62 ± 0.22
B-D-NaOH	1.54 ± 0.05	48.48 ± 0.59	19.44 ± 0.13

**Table 3:** Purity, number-average (M_n_) and weight-average (M_w_) molecular weights and polydispersity indexes (M_w_/M_n_) for the three lignin samples under study.

Lignin Sample	Purity (%)	M_n_ (g/mol)	M_w_ (g/mol)	M_w_/M_n_
L-NaOH	73.3	1649	11,009	6.68
L-AFW	83.5	3537	11,552	3.27
L-A180-AFW	92.0	3316	10,106	3.05

## Data Availability

The data presented in this study are available on request from the corresponding author.

## Abbreviations

Posidonia oceanica balls	POB
Autohydrolyzed raw material (130 °C)	A130
Autohydrolyzed raw material (150 °C)	A150
Autohydrolyzed raw material (180 °C)	A180
Acetoformosolv delignified autohydrolyzed raw material (180 °C)	D-A180-AFW
Alkali delignification of the raw material	D-NaOH
Acetoformosolv delignified raw material	D-AFW
Bleached alkali delignified raw material	B-D-NaOH
Bleached acetoformosolv delignified raw material	B-D-AFW
Bleached acetoformosolv delignified autohydrolyzed raw material (180 °C)	B-D-A180-AFW
CNF from bleached delignified autohydrolyzed raw material (180 °C)	CNF-A180-AFW
CNF from bleached alkali delignified raw material	CNF-NaOH
CNF from bleached acetoformosolv delignified raw material	CNF-AFW
Lignin from acetoformosolv delignification after autohydrolysis (180 °C)	L-A180-AFW
Lignin from acetoformosolv delignification	L-AFW
Lignin from alkali delignification	L-NaOH
